# Microbial diversity and composition in the gut microbiome of patients during systemic inflammatory response syndrome: can we use gut bacteria as potential biomarkers to characterize sepsis?

**DOI:** 10.3389/fcimb.2025.1622866

**Published:** 2025-08-22

**Authors:** Rafaela Ramalho Guerra, Patricia da Silva Fernandes, Otávio von Ameln Lovison, Giovanna de Ross Forni, Gabriel Silva de Oliveira, Luana Cristina Viana, Dariane Castro Pereira, William Latosinski Matos, Miriane Melo Silveira Moretti, Fabiana Zempulski Volpato, Luciana Giordani, Patricia Orlandi Barth, Tarsila Vieceli, Diego Rodrigues Falci, Márcio Manozzo Boniatti, Afonso Luís Barth, Andreza Francisco Martins

**Affiliations:** ^1^ Bacterial Resistance Research Laboratory (LABRESIS), Hospital de clínicas de Porto Alegre (HCPA), Experimental Research Center, Porto Alegre, Brazil; ^2^ Postgraduate Program in Pharmaceutical Sciences, Federal University of Rio Grande do Sul, Porto Alegre, Rio Grande do Sul, Brazil; ^3^ Bioinformatics Core, Hospital de Clínicas de Porto Alegre, Porto Alegre, Rio Grande do Sul, Brazil; ^4^ Infectious Diseases Department, Hospital de Clínicas de Porto Alegre, Porto Alegre, Brazil; ^5^ Postgraduate Program in Medical Sciences, Federal University of Rio Grande do Sul, Porto Alegre, Rio Grande do Sul, Brazil; ^6^ Department of Critical Care, Hospital de Clínicas de Porto Alegre, Porto Alegre, RS, Brazil; ^7^ Department of Biosciences, Federal University of Paraná - Palotina Sector, Palotina, Paraná, Brazil

**Keywords:** amplicon sequencing, biomarker, gut microbiota, sepsis, SIRS

## Abstract

**Background:**

Critically ill patients, including those with systemic inflammatory response syndrome (SIRS) and sepsis, frequently exhibit gut microbiota disruption due to physiological stress and broad-spectrum antimicrobial therapy (AT). Although antibiotics are essential for controlling infection, they can destabilize the gut microbiota and may contribute to poorer clinical outcomes. The characterization of the gut microbiota of these patients may inform microbiota-based interventions to mitigate antibiotic-induced dysbiosis.

**Objective:**

This study aimed to identify key bacterial taxa that distinguish sepsis from non-sepsis patients.

**Methods:**

A total of 89 stool samples (51 non-sepsis, 38 sepsis) were evaluated by amplicon sequencing the 16S rRNA gene to assess microbiota diversity and differential abundance. Samples were stratified by antibiotic exposure time: early AT (within 5th days of initiation) and prolonged AT (6th to 10th days). Additionally, patients were also grouped based on their AT: beta-lactam combined with other antimicrobial classes (BL-combined) and beta-lactam monotherapy (BL).

**Results:**

During early AT, alpha diversity (Shannon index) was significantly lower in sepsis patients compared to non-sepsis patients (2.48 *vs*. 3.0, *p = 0.01*), whereas no significant difference was observed after prolonged treatment (2.65 *vs*. 2.89, *p = 0.58*). Beta diversity analysis (Aitchison distance) revealed significant differences between groups early AT (PERMANOVA, *p = 0.005*), but not in the later phase (*p = 0.54*), suggesting that microbial communities converge over time. Early AT taxonomic profiling showed a decrease in *Anaerobutyricum* spp. and an increase in *Holdemania* spp. in the sepsis group. In the non-sepsis group, *Veillonella* spp. was impacted by time and beta-lactam combination. *Turicibacter* spp. showed a reduction in the prolonged AT sepsis group, while *Klebsiella* spp. was more abundant in the BL-combined sepsis patients.

**Conclusions:**

Sepsis and non-sepsis patients showed distinct gut microbiota profiles in early AT. In sepsis, the loss of taxa involved in key metabolic functions, as short-chain fatty acid production, reflects dysbiosis and may contribute to worse outcomes. Prolonged antibiotic use may favor enteropathogen overgrowth and gut translocation. These findings highlight the potential of microbiota-based strategies to guide antimicrobial therapy and improve clinical outcomes in critically ill patients.

## 1 Introduction

The intestinal microbiota is a symbiotic community with multiple functions. In a state of eubiosis, it plays an important role in the production of metabolites, including short-chain fatty acids (SCFAs) (Schulthess et al., 2019). It is also implicated in the defense against infection by opportunistic pathogens, regulates the immune response, and interacts closely with human cells to maintain homeostasis (Wozniak et al., 2022). Critical illness patients’ microbiomes may undergo an imbalance (dysbiosis), accompanied by endothelial damage resulting from an exaggerated host immune response (Dickson, 2016). ⁠This disruption diminishes the population of beneficial gut bacteria and increases intestinal permeability, facilitating the translocation of pathogens into the bloodstream and lymphatic system, which could be linked to sepsis. Consequently, this process exacerbates systemic inflammation and contributes to poor patient outcomes (Wozniak et al., 2022).

Sepsis is a life-threatening organ dysfunction resulting from a dysregulated host response to infection (Singer et al., 2016). Despite its well-established definition, distinguishing sepsis from other conditions remains challenging due to overlapping pathophysiologic features, which complicate bedside diagnosis (Póvoa et al., 2023).

Antimicrobial therapy is a fundamental component in the management of critically ill patients with suspected sepsis. The recommendation is initiating empiric broad-spectrum antibiotics within one hour of diagnosis, followed by de-escalation based on pathogen identification and susceptibility testing (Evans et al., 2021). However, the diagnostic process is complex, and delays remain a major contributor to increased morbidity and mortality (Rudd et al., 2020; Evans et al., 2021). Treatment duration typically ranges from 7 to 10 days but may vary depending on factors such as the source of infection, the causative pathogen, and the clinical patient’s response (Evans et al., 2021).

The beta-lactam class is the first line of antimicrobial therapy for the treatment of sepsis due to its broad-spectrum coverage of gram-positive and gram-negative bacteria (Evans et al., 2021; Novy et al., 2023). Moreover, the etiologic agent may not be identified (Sari et al., 2023), and the use of broad-spectrum antimicrobials for long periods, especially in immunocompromised patients, frequently represents the most viable therapeutic strategy (Strich et al., 2020; Kullberg et al., 2025).

Antimicrobials can generally alter the microbiome by killing commensal bacteria that produce peptides and metabolites related to gut health (Shah et al., 2021). This imbalance disrupts the normal functions of the gut microbiota, reducing resistance to colonization and facilitating the growth of harmful pathogens (Horrocks et al., 2023).

In sepsis, the cumulative burden of physiological stressors, sustained exposure to antimicrobials, artificial feeding, and hospitalization stay, can lead to a pathological cycle of dysbiosis, contributing to the worsening of the disease (Miller et al., 2021; Shahid et al., 2024). Consequently, the gut microbiome not only reflects a patient’s vulnerability but also represents a therapeutic target: microbiota-directed interventions have demonstrated potential to prevent or attenuate critical illness (Biemond et al., 2023; Fu et al., 2024; Han et al., 2024).

Therefore, this study aimed to characterize the diversity and composition of the intestinal microbiota in patients with Systemic inflammatory response syndrome (SIRS) to identify microbial taxa that distinguish between sepsis and non-sepsis cases. We also evaluated significant shifts in microbial taxa at two time points during antimicrobial therapy: within the first five days of treatment and after five days.

## 2 Methodology

### 2.1 Study design and enrolled patients

**Figure 1:**
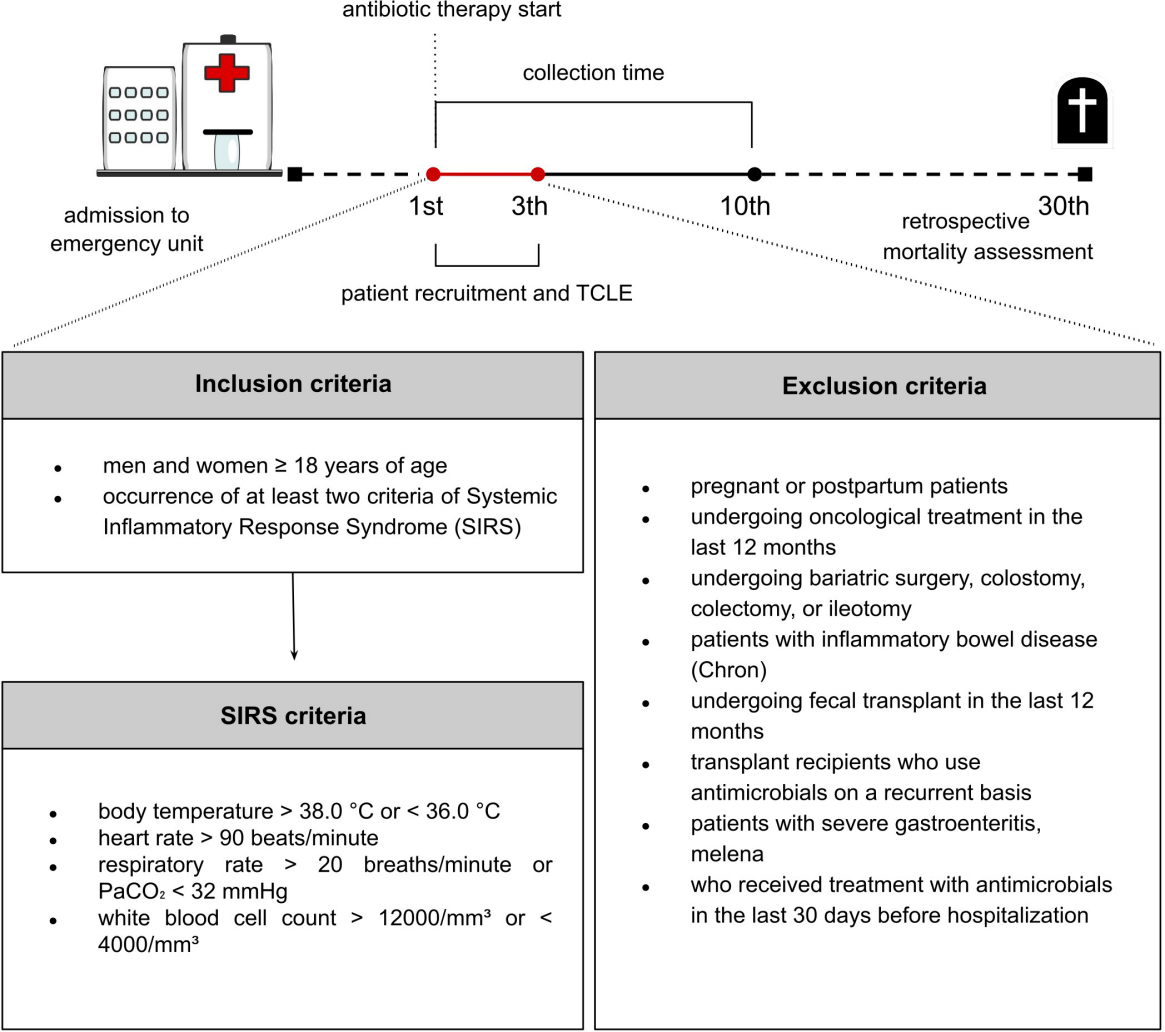
Timeline of the study follow-up and inclusion/exclusion criteria. Day 1st marks the initiation of antimicrobial therapy. Recruitment and the administration of the Free and Informed Consent Form (TCLE) were completed within the first 3 days of the study. Sample collection occurred up to the 10th day of antimicrobial therapy.

This observational cross-sectional study examines the diversity and composition of the gut microbiota in patients with SIRS. Patients were enrolled from the Emergency Department of Hospital de Clínicas de Porto Alegre (HCPA) between August 2022 and August 2023. Eligible participants had initiated intravenous antimicrobial therapy in the last 24 hours. All inclusion and exclusion criteria are described in (**Figure 1**).

Informed consent was obtained from all participants or their legal representatives, and patient data were handled in accordance with the Brazilian General Data Protection Law (LGPD). The first stool sample from each patient obtained after the initiation of antimicrobial therapy was included in the study. Samples collected after the tenth day of treatment were excluded. Demographic and clinical variables, including detailed antimicrobial therapy information, were extracted from the medical records (**Supplementary Tables S1**, **S2**). Participants were followed for 30 days to assess mortality outcomes.

For statistical and bioinformatic analysis, the patients were stratified into sepsis or non-sepsis groups. Sepsis was defined according to the SEPSIS-3 criteria (Singer et al., 2016), and the Sequential Organ Failure Assessment (SOFA) score was calculated within 24 hours of admission (Vincent et al., 1996). Patients with a SOFA score ≥ 2 were assigned to the sepsis group (**Supplementary Figure S1**), and all others to the non-sepsis group. To evaluate the impact of treatment duration on gut microbiota diversity, samples were categorized based on the timing of collection relative to antibiotic initiation: within 5 days (early therapy group) or between days 6 and 10 (prolonged therapy group). The exact number of days of antibiotic exposure at the time of sample collection is shown in **Supplementary Table S2**. Additionally, to better understand the impact of antibiotic treatment on the gut microbiota, all patients were categorized into two groups based on the prescribed antimicrobial therapy: beta-lactam in combination with any other antimicrobial class (BL-combined), and beta-lactam monotherapy (BL). This study was approved by the Research Ethics Committee (CEP) of the HCPA under registration number 58576722.2.0000.5327.

### 2.2 Specimen collection and DNA extraction

Stool samples were collected in sterile containers and kept refrigerated for transport. They were processed within 24 hours and stored at -80°C until further analysis.

Genomic DNA was extracted from 250mg of the sample using QIAamp PowerFecal Pro DNA Kits (Qiagen Inc., Germany). DNA concentration and purity were assessed using Nanodrop Lite Plus (Thermo Scientific) and then quantified using a Qubit DNA HS assay (Invitrogen) for genomic library preparation.

### 2.3 Amplicon sequencing of 16S rRNA

Genomic libraries were prepared using the 16S Metagenomic Sequencing Library Preparation Illumina^®^ using primers specific for the 16S rRNA V3-V4 region (460 bp) (Illumina, 2013). A negative control (molecular grade water) was included for quality assurance. Nextera XT Index Kit v2 Illumina was used for the DNA library preparation and PCR was performed with Phusion High-Fidelity PCR Master Mix with GC Buffer Enzyme (Invitrogen). All amplified PCR products were then cleaned using AMPure XP beads (Beckman Coulter). The library concentrations were quantified using a Qubit DNA HS assay (Invitrogen) and verified for fragment distribution by a capillary electrophoresis instrument 4200 TapeStation System (Agilent). Pooled libraries were loaded onto the MiSeq^®^ platform using a v3 Reagent Kit (2x300bp; ~ 200.000 reads/sample) (Illumina, Inc.).

### 2.4 Statistical analysis

The study population was characterized using demographic and clinical variables. Categorical variables were summarized as frequencies and percentages. The Wilcoxon test was employed to assess differences in continuous variables between study groups. The alpha diversity was performed using the Shannon index, followed by the Wilcoxon rank sum test. Furthermore, a linear regression model was employed to assess the impact of other variables on the diversity of microbiomes. The statistical significance and proportion of explained variance were assessed by permutational multivariate analysis of variance (PERMANOVA) (Anderson, 2017). The taxonomic differential abundance analysis was performed using the Analysis of Compositions of Microbiomes with Bias Correction 2 (ANCOM-BC2) (RRID: SCR_024901) algorithm (Lin and Peddada, 2023). Covariates, such as age, sex and exposure time of antimicrobial therapy, were incorporated into the modeling process and utilized for beta diversity calculations and differential abundance analysis.

### 2.5 Bioinformatic analysis

The bioinformatic analysis were performed in the Bioinformatics Core of HCPA using were performed with the open-source software R Project for Statistical Computing (RRID: SCR_001905) v. 4.5.0, the development interface RStudio (RRID: SCR_000432) v. 4.5.0, and packages of the project Bioconductor (RRID: SCR_006442) v. 3.21. Raw sequences were imported to the DADA2 (RRID: SCR_023519) to generate an amplicon sequence variant (ASV) table (Callahan et al., 2016). Reads were quality-checked, trimmed, filtered, and truncated according to quality plots. Paired-end joining, determination of ASV, and removal of chimeric sequences were performed, followed by the taxonomic assignment using the SILVA database RRID: SCR_006423 v.138.2.

The beta diversity analysis was conducted to evaluate the structural composition of the intestinal microbiome between groups (sepsis and no-sepsis). The non-metric multidimensional scaling (NMDS) of both Aitchison and Jaccard distance measures was performed to assess the structural composition of the microbiome (Jaccard, 1912; Aitchison et al., 2000).

## 3 Results

### 3.1 Study framework and recruited participants

**Figure 2:**
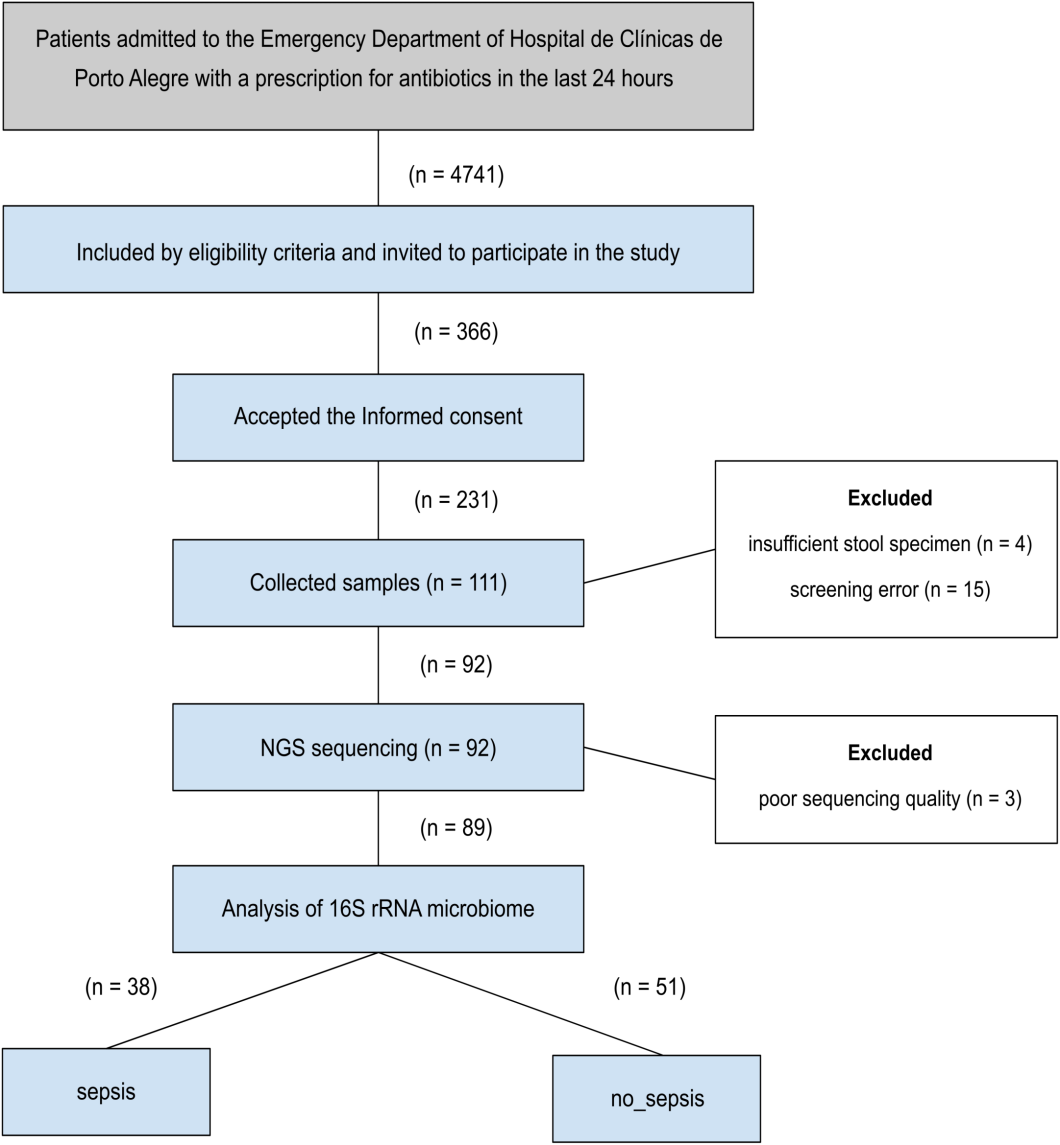
Flowchart of the study design and patient enrollment. The diagram illustrates the distribution of patients during recruitment, specimen collection, and sample processing for inclusion in the study.

A total of 4,741 patients were screened, and 366 met the inclusion criteria and were invited to participate in the study (**Figure 2**). Among them, 231 signed the informed consent form, and 111 provided stool samples. However, 19 patients were excluded due to insufficient sample quantity or screening errors.

Of the 92 samples submitted for sequencing, 89 met the quality criteria (Q > 30 for at least 50% of the total reads) and were analyzed for gut microbiota diversity and composition. A total of 38 patients were included in the sepsis group, while 51 were included in the non-sepsis group (**Figure 2**).

According to the specific time point of sample collection, 55 patients were classified as first five days of antimicrobial treatment (sepsis: 19; non-sepsis: 36), while 34 patients provided stool samples after the fifth day (up to ten days; sepsis: 19; non-sepsis: 15) (**Table 1**).

**Table 1:** Characteristics of sepsis and non-sepsis patients.

Variable/Outcome	Sepsis n = 38	Non-sepsis n = 51	*p-value*
Demographics			
Age mean (SD)	65.29 (15.59)	62.07 (17.42)	*0.371*
Male	24 (63.15%)	21 (41.17%)	*0.040*
Severity of disease			
SOFA score mean (SD)	4.32 (1,85)	0.58 (0.49)	*< 0.001*
Beta-lactam combination	23 (61.00%)	28 (55.00%)	*0.600*
Length of hospitalization mean (SD)	25.89 (18.15)	17.96 (27.01)	*0.009*
Length of antimicrobial therapy mean (SD)	18.16 (11.97)	10.23 (5.20)	*0.001*
30-day mortality	6 (15.78%)	3 (5.88%)	*0.162*
Collection specimens			
Before the 5th day of antimicrobial therapy	19 (50%)	36 (71%)	*0.021*
After the 5th day of antimicrobial therapy	19 (50%)	15 (29%)	*0.490*

SD, Standard deviation; SOFA, Sequential Organ Failure Score.

### 3.2 Patient demographics and clinical features

Demographic data and clinical characteristics of sepsis (n = 38) and non-sepsis patients (n = 51) are presented in **Table 1**. During the follow-up period, patients in the sepsis group had a significantly longer length of hospital stay (mean:25 days; DS: 18) compared to those in the non-sepsis group (mean:17 days; SD:27). Thirty-day intrahospital mortality did not differ significantly between the groups (*p = 0.162*). Additionally, patients with sepsis received a longer duration of antimicrobial therapy (mean: 18 days; SD: 12 vs. 10 days; SD: 5) (p = 0.001).

An analysis of antibiotic prescriptions administered during the collection time sampling (within ten days of antimicrobial therapy) revealed that Piperacillin-Tazobactam (TZP) was the most commonly prescribed antibiotic in the sepsis group (15/38; 39.47%) (**Supplementary Figure S2**). Moreover, 61.00% (23/38) of patients in this group received beta-lactams in combination with antibiotics from other classes (**Table 1**), with the most common being a combination of beta-lactams and glycopeptides (26.31%) (**Supplementary Figure S2**).

Conversely, in the non-sepsis group, Amoxicillin/Clavulanic Acid (AMC) was the most frequently used antibiotic (25/51; 49.01%). In this group, 55.00% (28/51) of patients received beta-lactams in combination with antibiotics from other classes (**Table 1**), with the most commonly prescribed combination including beta-lactams and macrolides (25.49%) (**Supplementary Figure S2**).

### 3.3 Comparison of sepsis and non-sepsis patients by gut microbiota

The sequencing of 89 stool samples yielded 13,147,925 filtered reads, which were clustered into 23,406 amplicon sequence variants (ASVs). Collinearity among variables and the presence of outliers in taxon distributions were observed. One sample was excluded from further analysis due to its identification as an outlier, resulting in a final sample size of n = 88.

**Figure 3:**
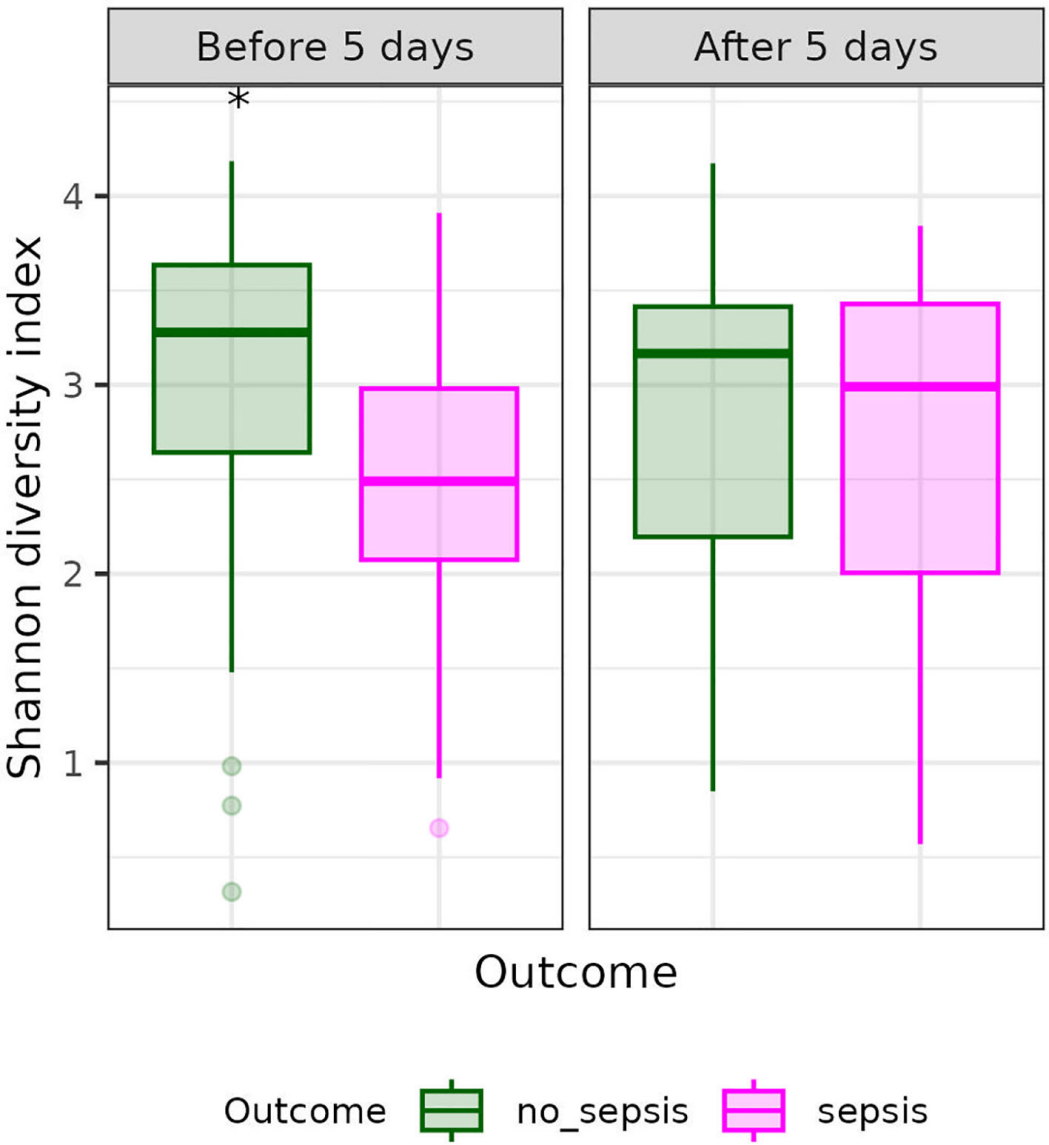
Alpha diversity in sepsis and non-sepsis groups at two time points during antimicrobial therapy. Alpha diversity, measured by the Shannon index, was compared between groups before the fifth day of antimicrobial therapy (sepsis: 3.0 *vs*. non-sepsis: 2.48, *p = 0.01*) and after the fifth day (sepsis: 2.89 *vs*. non-sepsis: 2.65, *p = 0.58*). The sepsis group is shown in purple, and the non-sepsis group in green.

The non-sepsis group exhibited greater alpha diversity than the sepsis group (Shannon index: 2.97 *vs*. 2.56, *p = 0.02*) (**Supplementary Figure S3**). Additionally, for two times of antimicrobial therapy, before the fifth day, the alpha diversity was significantly higher in the non-sepsis group compared to the sepsis group (Shannon index: 3.0 *vs*. 2.48, *p = 0.01*). However, after the fifth day of antimicrobial therapy, no significant difference in alpha diversity was observed between the groups (Shannon index: 2.89 *vs*. 2.65, *p = 0.58*) (**Figure 3**).

**Figure 4:**
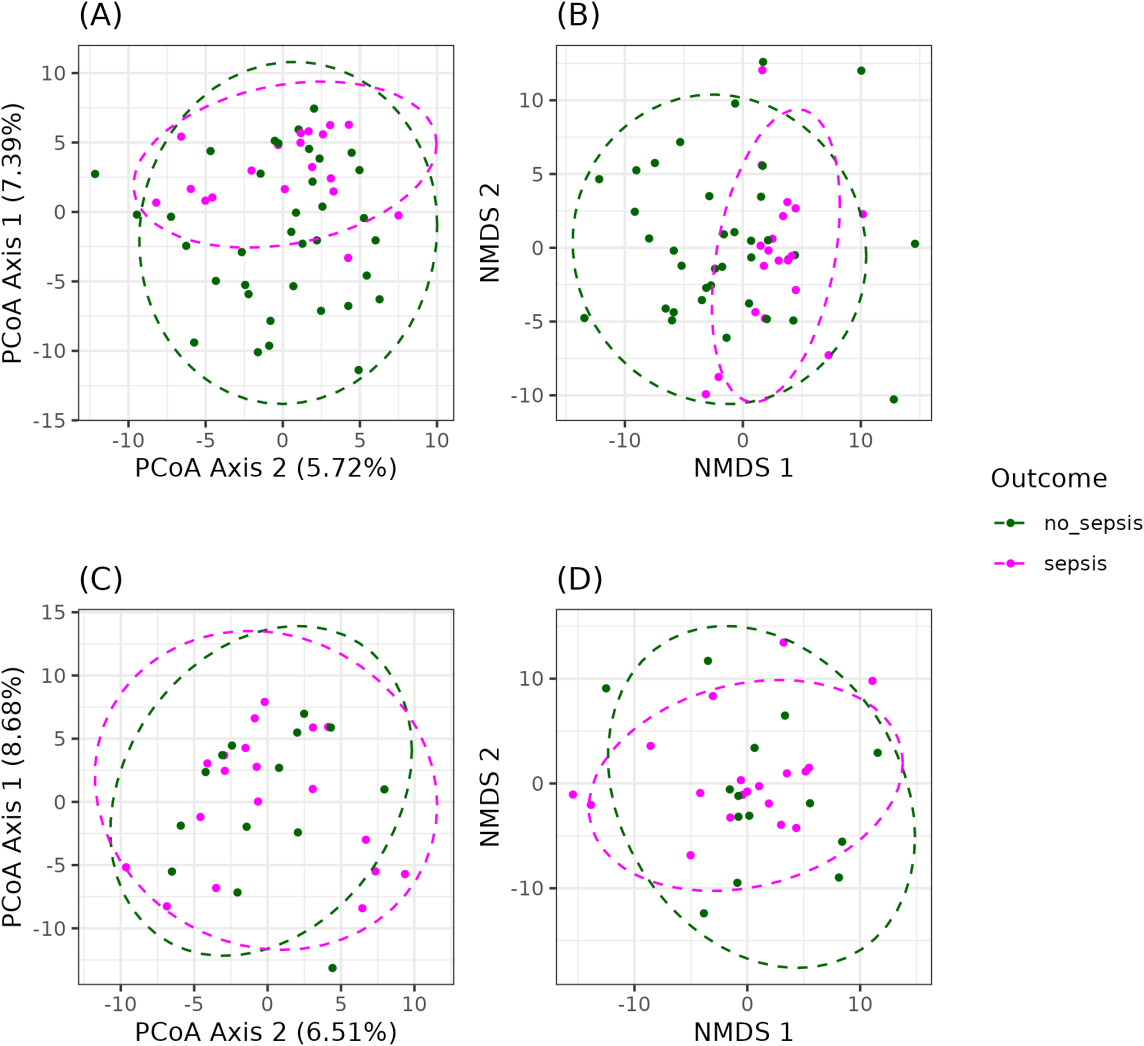
Beta diversity between sepsis and non-sepsis groups at two time points during antimicrobial therapy based on Aitchison distance. **(A)** Principal Coordinate Analysis (PCoA) and **(B)** Non-metric multidimensional scaling (NMDS) plots before the fifth day of antimicrobial therapy. **(C)** PCoA plots and **(D)** NMDS plots after the fifth day of therapy. Sepsis patients are represented in purple and non-sepsis patients in green. Statistical significance (adonis2, *p = 0.004*) and the proportion of explained variance were assessed using permutational multivariate analysis of variance (PERMANOVA).

Analysis of dissimilarities showed significant differences between the sepsis and non-sepsis groups for Aitchson distance (**Supplementary Figures S4A, B**; adonis2, *p = 0.001*), and Jaccard distance (**Supplementary Figures S4C, D**; adonis2, *p = 0.002*). When stratified by duration of antimicrobial therapy, significant differences in Aitchison distance were observed among patients within the first five days of treatment (**Figures 4A, B**; adonis2, *p = 0.005*). However, no significant differences were detected between the groups after five days of antimicrobial therapy (**Figures 4C, D**; adonis2, *p = 0.57*). The relative abundance results showed that Bacillota and Bacteroidota were the most abundant phyla among the patients (**Supplementary Figure S6A**). Lachnospiraceae and Ruminococcaceae were the most abundant families (**Supplementary Figure S6B**), while the genera more observed were *Bacteroides* spp. and *Blautia* spp., particularly in the sepsis group (**Supplementary Figure S6C**).

### 3.4 Antimicrobial therapy time and differential abundance between groups

**Figure 5:**
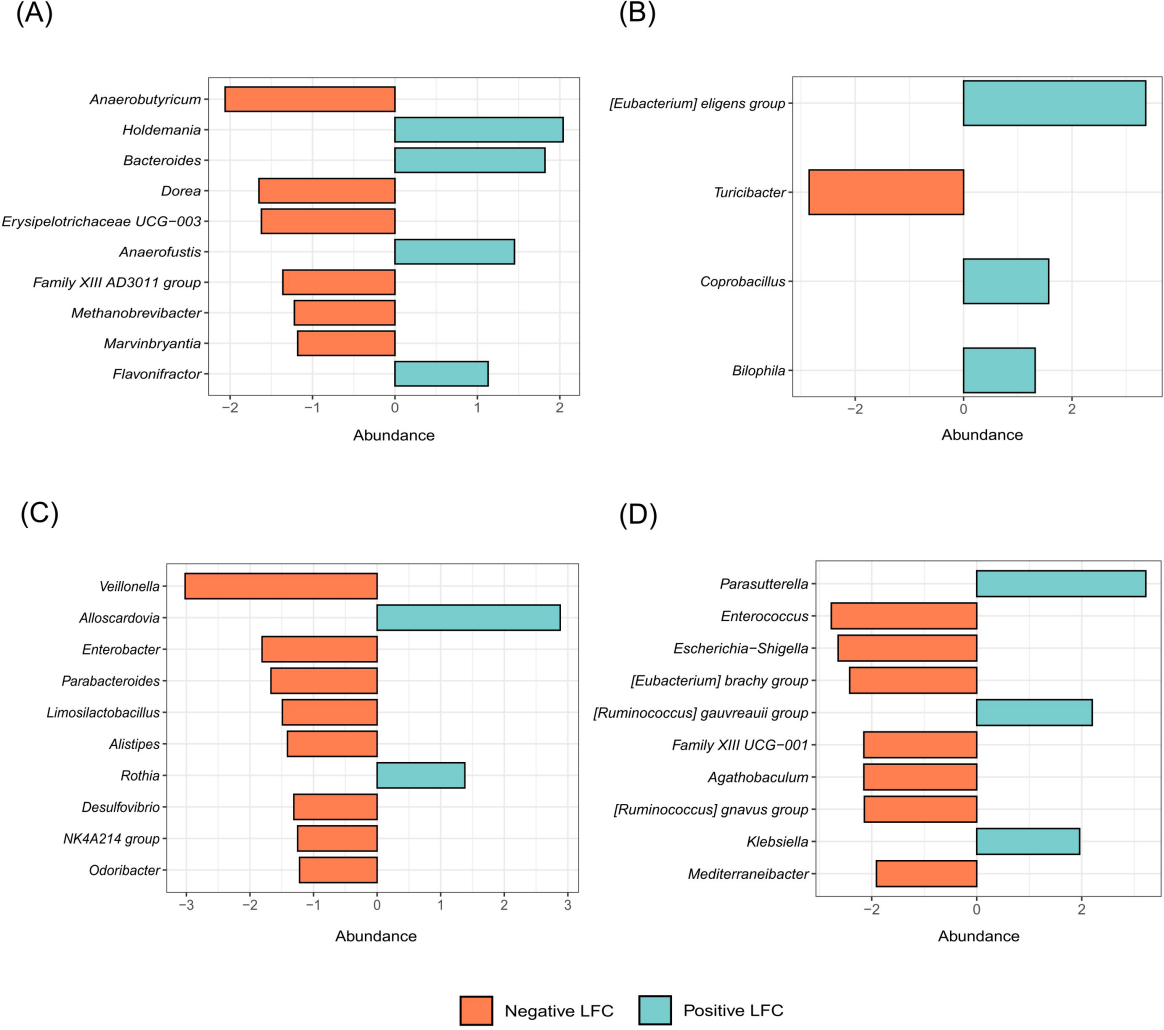
Bar chart showing the top 10 differentially abundant microbial taxa stratified for outcome, antimicrobial therapy type and two time points of treatment. **(A)** Differential abundance of taxa between the sepsis and non-sepsis groups before the fifth day of treatment (early therapy). **(B)** Differential abundance between the same groups after the fifth day of treatment (prolonged therapy). **(C)** Comparison of taxa abundance in patients receiving a combination of beta-lactams and other antimicrobial classes versus those receiving beta-lactam monotherapy, during early therapy. **(D)** Same comparison during prolonged therapy. The x-axis shows the log-fold change (LFC) in abundance, while the y-axis represents taxa annotated at the genus level. Blue bars indicate genera with higher abundance, and orange bars represent genera with lower abundance.

A differential abundance of microbial taxa was observed between outcome groups. During the first five days of antimicrobial therapy, a reduction in *Anaerobutyricum* spp. and an increase in *Holdemania* spp. were noted in the sepsis group (**Figure 5A**). After day five, the sepsis group showed a decline in the genus *Turicibacter* spp., whereas members of the *Eubacterium* group showed increased abundance (**Figure 5B**).

When evaluating the type of antimicrobial therapy (BL-combined ***vs***. BL), differences in microbial composition were also observed. A marked reduction in the genus *Veillonella* spp. was observed in patients receiving BL-combined therapy during the early phase of treatment (**Figure 5C**). After five days of therapy, patients in the BL-combined group demonstrated a broader reduction in several genera, including *Escherichia-Shigella* spp. and other gram-positive taxa (**Figure 5D**).

### 3.5 Antimicrobial therapy time and differential abundance stratified by groups

In the sepsis group, BL-combined patients exhibited an increased abundance of the genus *Klebsiella* spp. (**Supplementary Figure S5A**). Additionally, taxa most affected by each additional day of antimicrobial therapy included a reduction in *Agathobacter* spp. and an increase in *Massiliomicrobiota* spp. (**Supplementary Figure S5B**). In contrast, among non-sepsis patients, no genus was found to be increased in those receiving beta-lactam combination therapy. However, a marked decrease in the genus *Veillonella* spp. was observed (**Supplementary Figure S5C**), consistent with the pattern previously identified without stratification by clinical outcome (**Figure 5C**). The duration of antimicrobial therapy in non-sepsis patients appeared to influence several genera, most notably a decrease in *Veillonella* spp. and an increase in *Extibacter* spp. (**Supplementary Figure S5D**).

## 4 Discussion

### 4.1 Diversity of gut microbiota along antimicrobial therapy

Patients suffering from sepsis who are exposed to long-term antimicrobial therapy have been associated with a decrease in the diversity in the gut microbiome and an increase in taxa with pathogenic potential (Pettigrew et al., 2019; Liu et al., 2020; Luan et al., 2024). In this study, it was observed that the intestinal microbiota of patients with sepsis and non-sepsis differs significantly, particularly during the initial days of antimicrobial therapy. After the fifth day of antimicrobial therapy, a decline in alpha diversity was observed in both groups, suggesting that, despite their initially distinct characteristics, their microbial compositions may converge over time.

Beta-lactams are the most commonly used antibiotics in sepsis treatment, a trend also observed in this study (Roberts and Lipman, 2009; Novy et al., 2023). These antibiotics have a broad spectrum of activity and demonstrate superior tissue penetration compared to other antimicrobial classes, such as aminoglycosides (Blot et al., 2014; Niederman et al., 2021). TZP was the beta-lactam most commonly prescribed antimicrobial in the sepsis group. Long-term TZP treatment has been shown to reduce serum cytokine levels, modulate the immune response (Wang et al., 2022), promote *Enterococcus* dominance (van der Waaij et al., 1971; Kullberg et al., 2021), and decrease the relative abundance of *Bacteroidota*, with only gradual recovery following treatment cessation (Leopold et al., 2022).

Antimicrobial therapy can promote the development of resistance (Xu et al., 2020) and act as a driver of nosocomial infections (Cho et al., 2024), with gastrointestinal dysbiosis contributing to poor clinical outcomes. Moreover, the combination of beta-lactams with other classes of antibiotics, such as glycopeptides and macrolides, in patients with sepsis and non-sepsis, may be indicative of a decrease in genera belonging to the Firmicutes phylum due to their broad action on gram-positive bacteria. A reduction in Firmicutes has been associated with increased mortality in septic intensive care unit (ICU) patients (Bhalodi et al., 2019). Therefore, the combination of classes and the duration of therapy should be periodically discussed and reassessed in patients with sepsis.

### 4.2 Gut microbiota and cross-talk metabolic health

Loss of beneficial microbes and overgrowth of pathogens compromise gut integrity and alter microbial metabolite profiles, affecting distant organs through the gut-organ axis. This cross-talk contributes to disease progression and complicates the management of critically ill patients (Agudelo-Ochoa et al., 2020; Wozniak et al., 2022). These metabolic alterations may result from both the disease’s pathophysiology and the depletion of commensal bacteria responsible for producing SCFAs and secondary bile acids (Gipson et al., 2020; Wozniak et al., 2022; Ahmad et al., 2025).

In the first days of antimicrobial therapy, *Anaerobutyricum* spp. is decreased in the gut microbiome in sepsis patients. This genus belongs to the *Lachnospiraceae* family and some species can produce butyrate (SCFA) from lactate and acetate, even in acidic environments, being a taxon with potential probiotic use (Duncan et al., 2004). Butyrate serves as an energy source for colonocytes, which are responsible for maintaining the balance of intestinal permeability (Canani et al., 2011; Hodgkinson et al., 2023). Although there are no studies related to sepsis, this genus has already been associated with the potential modulation of glucose metabolism, being a key taxon in patients with metabolic syndrome (Wortelboer et al., 2022; Hutchison et al., 2024). Furthermore, *Anaerobutyricum* spp. was not observed to be differentially abundant among patients receiving BL-combined, nor were temporal shifts detected. Consequently, the depletion of this genus in sepsis patients during the initial days of antimicrobial therapy could serve as an indicator of increased risk for early disease severity, attributable to its protective role.

In contrast, *Holdemania* spp. was initially found to be more abundant in the gut microbiota of patients with sepsis. This genus has also been associated with conditions such as irritable bowel syndrome and cardiovascular disease (Dai et al., 2023). Notably, *Holdemania* spp. plays a key role in degrading mucin, a major component of the mucus layer that protects the intestinal epithelium (Raimondi et al., 2021). Although a reduction in *Holdemania* spp. was observed among BL-combined sepsis patients, the initial increase in this genus is a notable finding. This finding suggests a potential predisposition to systemic inflammatory responses driven by an intestinal environment with compromised barrier integrity (Dickson, 2016).

Long-term antimicrobial therapy results in the gut microbiome being more similar and less diverse. However, the *Turicibacter* spp. showed decreased abundance in patients with sepsis after the fifth day of antimicrobial therapy. This is curious since this genus belongs to the *Erysipelotrichaceae* family and has been associated with its ability to modulate the immune response and serotonin signaling, both of which are closely linked to intestinal inflammation and cancer development (Kaakoush, 2015; Karmakar and Lal, 2021; Hamada et al., 2023; Lin et al., 2023). Moreover, species within this genus have been linked to lipid and bile acid metabolism (Lynch et al., 2023).

The microbial metabolism plays a critical role in regulating the immune system (Takeuchi et al., 2024). Sepsis is known to impair the production of SCFAs and secondary bile acids (Long et al., 2023). In contrast, septic patients with liver dysfunction may exhibit exacerbated bile acid production (Shahid et al., 2024). Alterations in bile acid concentrations in septic patients may reflect changes in the abundance of intestinal bacteria capable of metabolizing these compounds (Kaakoush, 2015; Nakov et al., 2020; Karmakar and Lal, 2021; Zhang et al., 2022; Shahid et al., 2024). Thus, the decline in *Turicibacter* spp. may contribute to metabolic imbalances and immune dysregulation during sepsis. Consequently, this taxon emerges as a key taxon for understanding the dynamics of the intestinal microbiota during immune response dysregulation in patients with sepsis.

In addition, the decrease in SCFA producers and bile acid metabolizers from the *Lachnospiraceae* and *Ruminococcaceae* families, before and after the fifth day of antimicrobial therapy, highlights changes in the intestinal microbiota and the loss of key commensal bacteria involved in metabolism.

Regarding patients who received a combination of beta-lactams with other classes of antimicrobials, we observed that the *Veillonella* spp. was decreased in the initial days of therapy. This effect is particularly pronounced in the non-sepsis group. This genus plays a key role in degrading lactate into propionate and acetate (SCFAs). Lactate degradation in the intestine contributes to maintaining pH levels by preventing acidification of the medium (Zhang et al., 2024). Reduced *Veillonella* spp. abundance may allow for the growth of acid-tolerant enteropathogens and microbial translocation due to epithelial damage (Louis et al., 2022). These findings suggest that although non-sepsis patients initially present with a more diverse gut microbiota, it is rapidly affected by the use of BL-combined, potentially increasing their susceptibility to more severe clinical outcomes over time.

Conversely, after the fifth day of antimicrobial therapy, an increased abundance of *Klebsiella* spp. was observed between outcome groups who received prolonged combination antimicrobial therapy. This result was observed mainly in patients with sepsis. One study found that approximately 50% of *Klebsiella pneumoniae* infections in intensive care unit (ICU) patients originated from their intestinal microbiota (Gorrie et al., 2017). Since antimicrobial therapy can promote the spread of antimicrobial resistance genes (ARGs) in the intestine, the translocation of resistant enteropathogens in sepsis patients is concerning (Ahmad et al., 2025). Infections caused by bacteria of this genus are difficult to treat because of their ability to produce biofilms and acquire and disseminate multiple ARGs, including those that confer resistance to carbapenems, which are used as a last resort in the treatment of sepsis (Martin and Bachman, 2018). Our results underscore the importance of identifying taxa related to worsening manifestations in sepsis patients.

Interestingly, our findings are promising for the use of gut microbiota modulation, personalized medicine, and stewardship of antimicrobials in sepsis treatment. However, it is important to note that this study is subject to certain limitations. The sequencing techniques used in this study allow for bacterial identification down to the genus level. However, approaches involving whole 16S rRNA gene sequencing or genomic shotgun approach could enable species-level resolution, which may provide a more detailed understanding of the role of the intestinal microbiota in sepsis. Additionally, the number of samples collected in the initial days following admission was relatively limited, which precludes any meaningful conclusions regarding the initial differences in the microbiota of these patients and the potential impact of the length of hospital stay and antimicrobial therapy. The dietary type and body mass index (BMI) of the patients were not considered in the analysis of microbiota composition. Furthermore, the classification of sepsis using the SOFA score can be misleading and may not always accurately reflect the patient’s true condition. A classification approach based on machine learning may provide a more precise method for distinguishing these patients according to their microbiota profiles.

On the other hand, our study is particularly relevant as it compares the gut microbiota of patients with sepsis to non-septic patients exhibiting SIRS symptoms. Observing differences in the microbiota within the first days of antimicrobial therapy between these two groups may suggest that, despite their clinical similarities, they harbor distinct microbial taxa. The microbial taxa identified in this study may serve as potential biomarkers of disease severity in SIRS patients. However, further research is necessary to characterize these taxa in terms of their functional roles and interactions with the host.

## Data Availability

The datasets presented in this study can be found in online repositories. The names of the repository/repositories and accession number(s) can be found below: https://www.ncbi.nlm.nih.gov/, PRJNA1255639.
