# Supplementary material for: Microbial diversity and composition in the gut microbiome of patients during systemic inflammatory response syndrome: can we use gut bacteria as potential biomarkers to characterize sepsis?
Source: Front Cell Infect Microbiol. 2025 Aug 22;15:1622866. doi: 10.3389/fcimb.2025.1622866 (PMC12411461; doi:10.3389/fcimb.2025.1622866)
Supplement: Supplementary file 1 [file DataSheet1.docx]

***Supplementary Material***


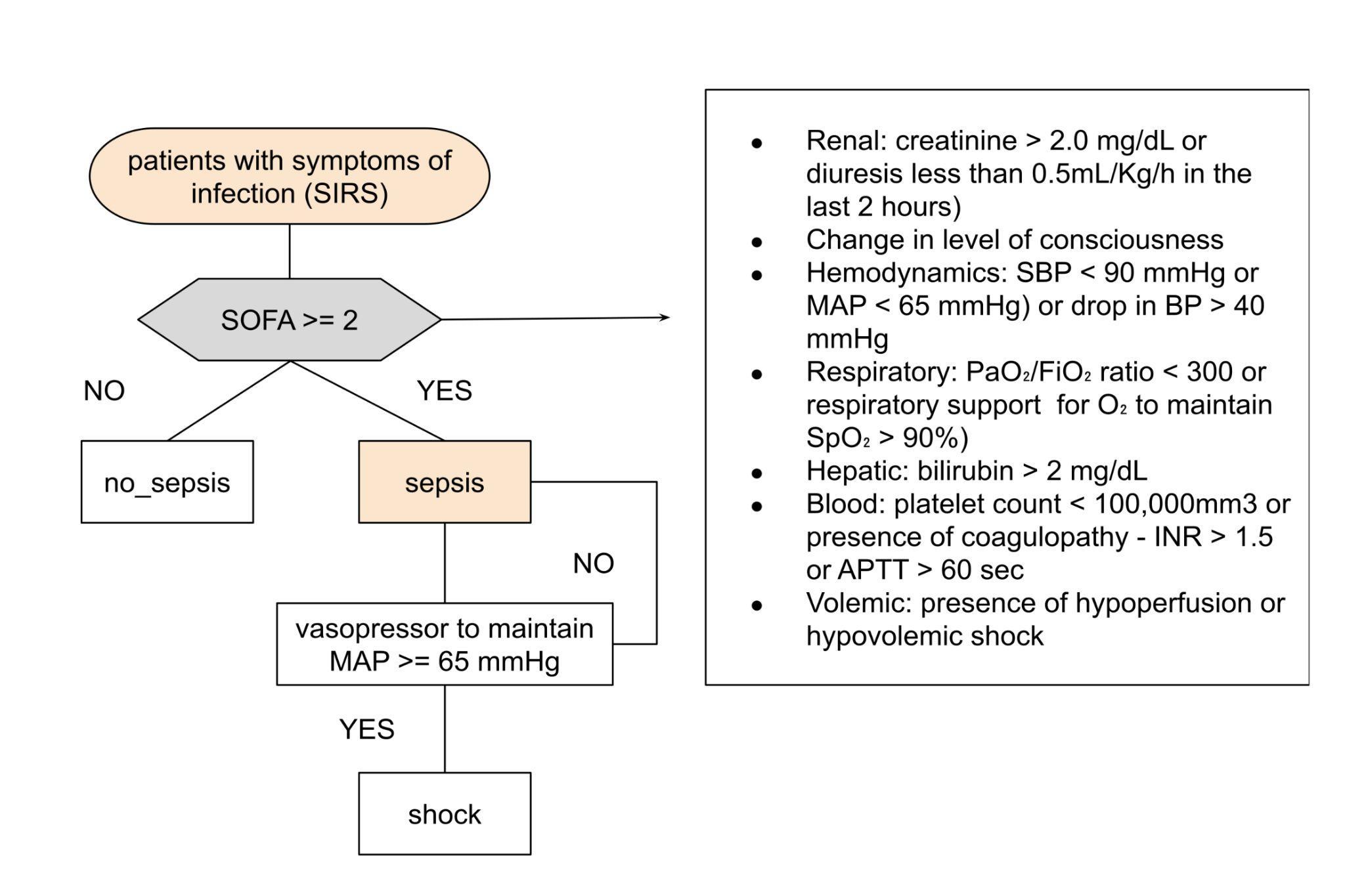


**Supplementary Figure 1.** **Flowchart illustrates the classification of sepsis patients based on the SOFA score and the identification of septic shock.** The diagram outlines the criteria used to assign patients to the sepsis group according to Sequential Organ Failure Assessment (SOFA) scoring and further classification for septic shock.


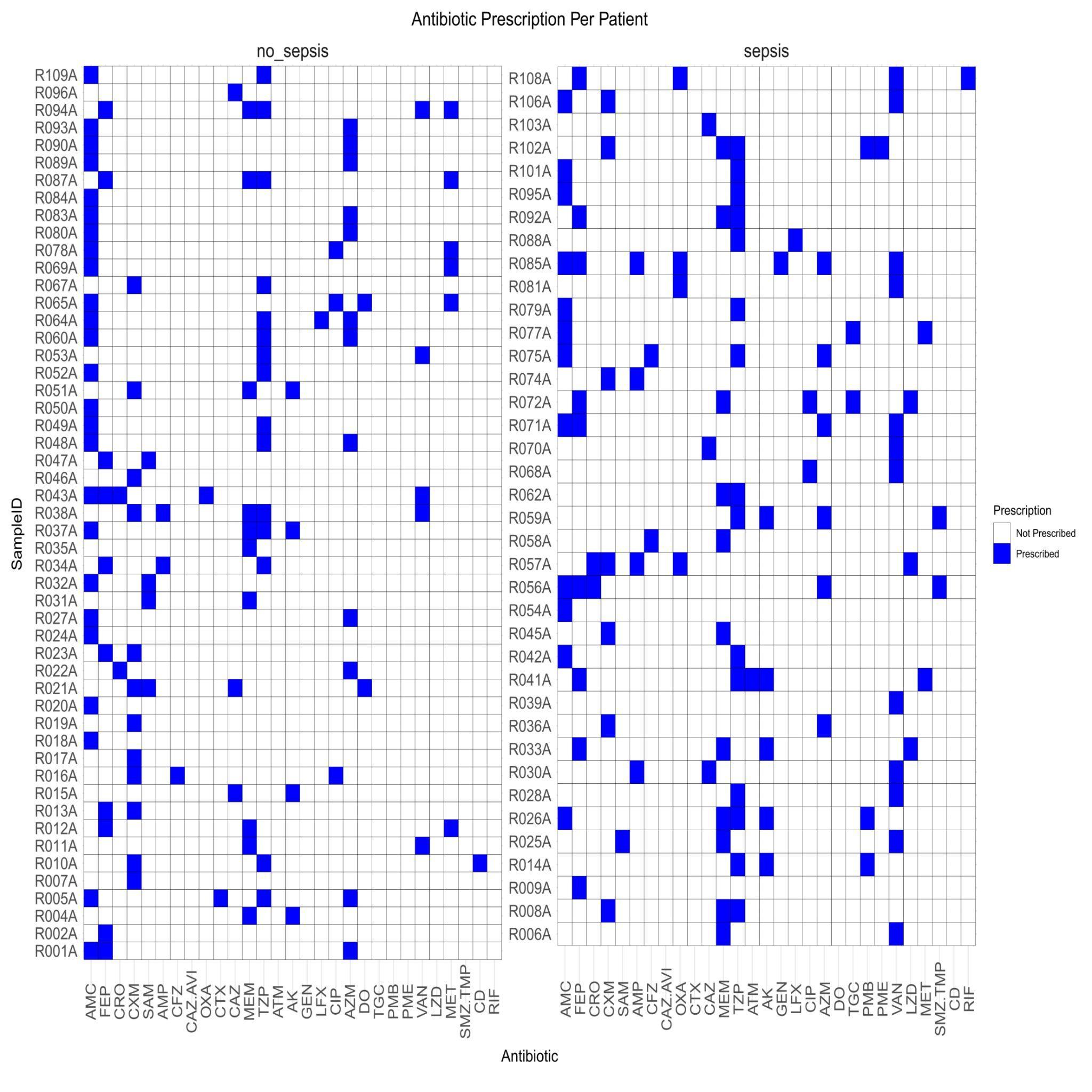


**Supplementary Figure 2.** **Tile plot of antimicrobials prescribed before fecal sample collection in non-sepsis and sepsis groups.** The left panel represents patients in the non-sepsis group, and the right panel represents those in the sepsis group. The y-axis displays patient IDs within each group, and the x-axis shows antimicrobial agents and the number of patients who received each. AMC, Amoxicillin/Clavulanic Acid; FEP, Cefepime; TZP, Piperacillin/Tazobactam; LFX, Levofloxacin; CRO, Ceftriaxone; MEM, Meropenem; MET, Metronidazole; AK, Amikacin; PMB, Polymyxin B; CXM, Cefuroxime; SAM, Ampicillin/Sulbactam; DO, Doxycycline; AZM, Azithromycin; SMZ, Sulfamethoxazole (commonly with trimethoprim as SMZ-TMP); VAN, Vancomycin; CD, Clindamycin; LZD, Linezolid; AMP, Ampicillin; CFZ, Cefazolin; CAZ/AVI, Ceftazidime/Avibactam; ATM, Aztreonam; OXA, Oxacillin; CTX, Cefotaxime; GEN, Gentamicin; PME, Polymyxin E (Colistin); CAZ, Ceftazidime; RIF, Rifampin.

**
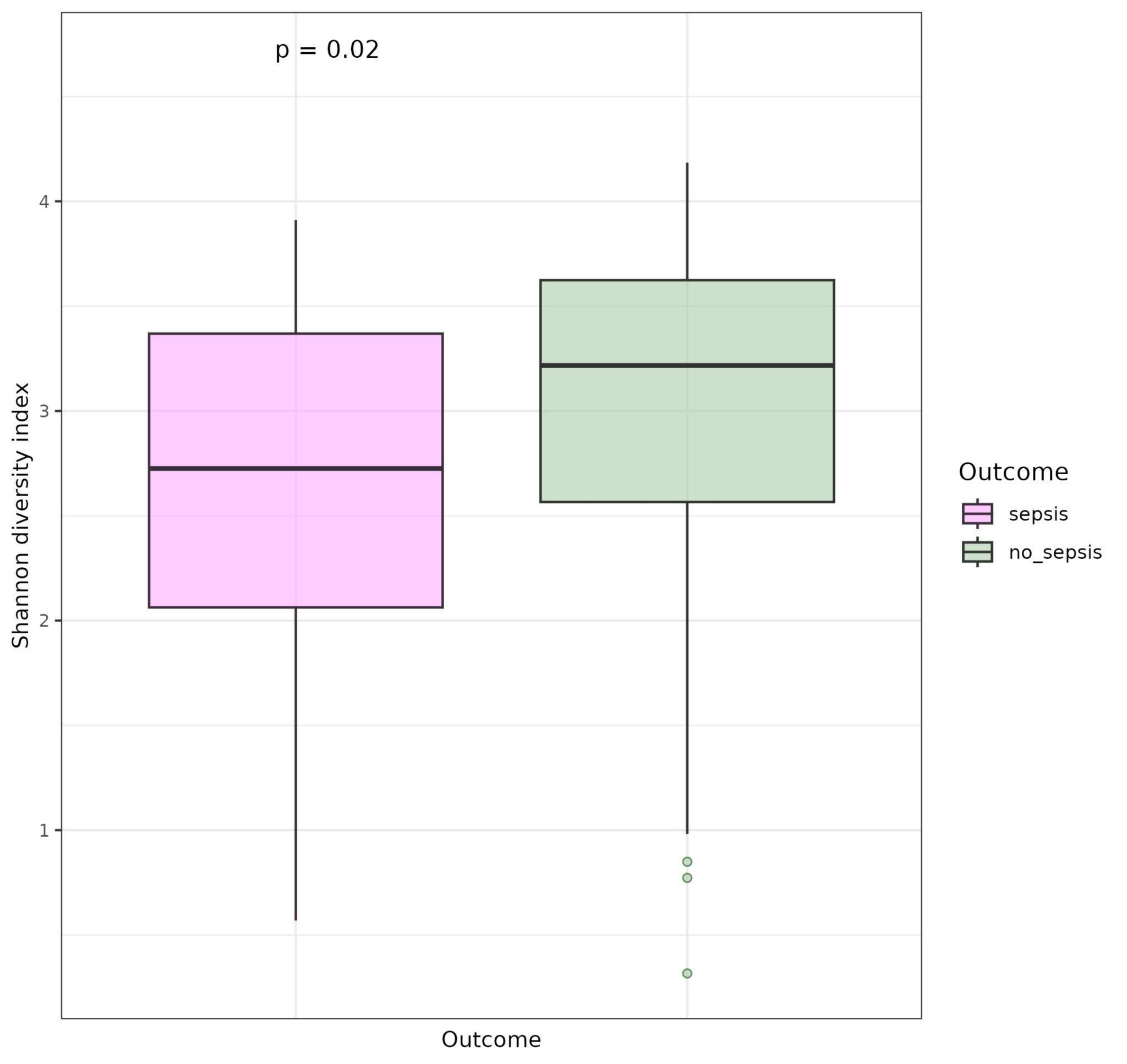
**

**Supplementary Figure 3.** **Alpha diversity between sepsis and non-sepsis groups based on the Shannon index.** The sepsis group exhibited significantly higher alpha diversity compared to the non-sepsis group (Shannon index: 2.97 vs. 2.56, p = 0.02). The sepsis group is shown in purple, and the non-sepsis group in green.


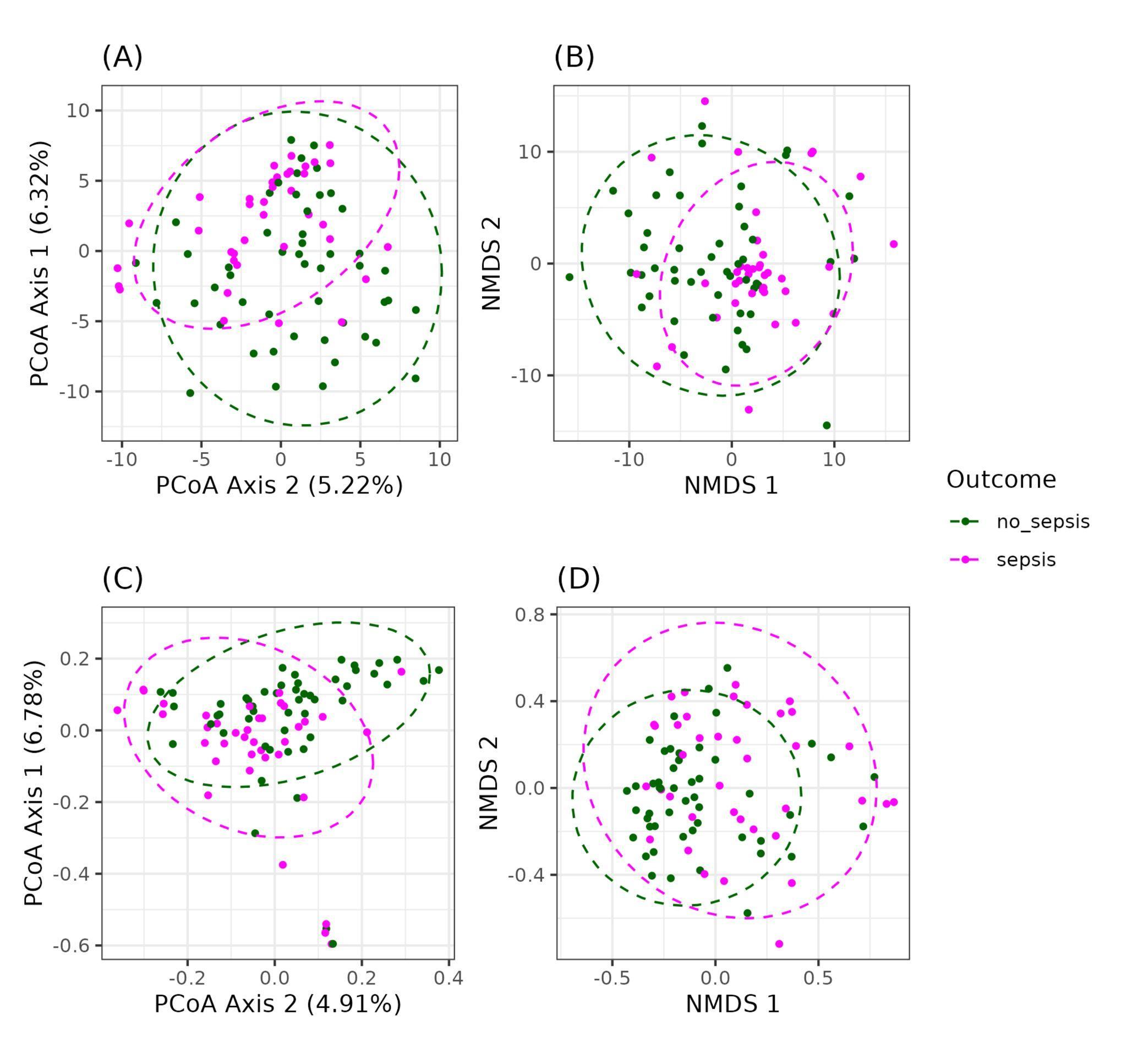


**Supplementary Figure 4. Beta diversity between sepsis and non-sepsis groups based on Aitchison and Jaccard distances. (A)** and **(B)** Principal Coordinate Analysis (PCoA) and non-metric multidimensional scaling (NMDS), respectively, based on Aitchison distance (adonis2, p = 0.004). **(C)** and **(D)** PCoA and NMDS based on Jaccard distance (adonis2, p = 0.001). The sepsis group is shown in purple and the non-sepsis group in green.


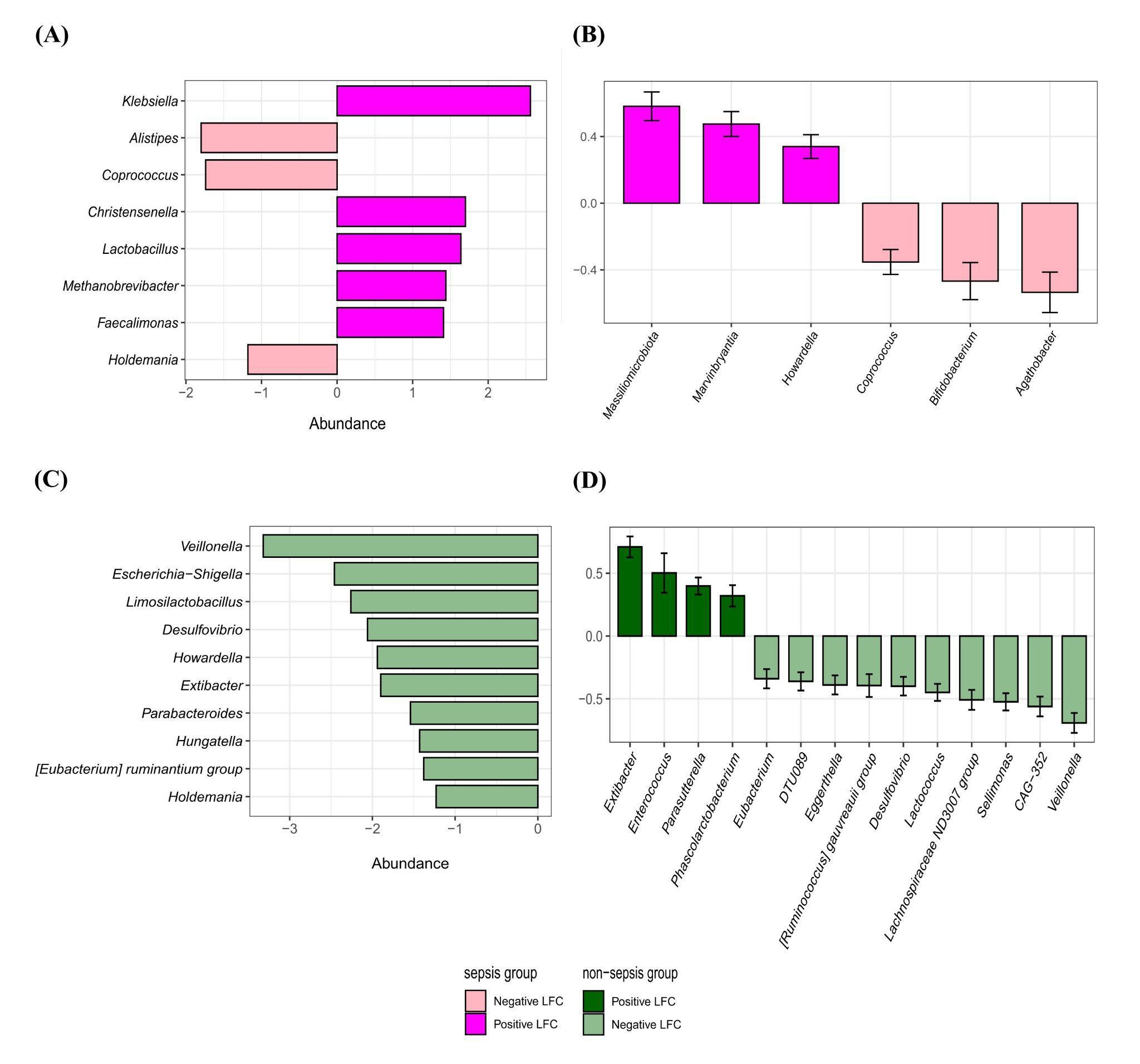


**Supplementary Figure 5. Differential abundance of microbial taxa by type and duration of antimicrobial therapy, stratified by outcome group. (A)** Differential abundance between sepsis patients that received beta-lactam combined with other antimicrobial classes (BT-combined) vs. patients that receive beta-lactam monotherapy (BT) and **(B)** Differential abundance over time of antimicrobial therapy (AT) in sepsis patients. **(C)** Differential abundance between non-sepsis BT-combined patients vs. BT patients and **(D)** The x-axis shows the log-fold change

(LFC) in abundance, while the y-axis represents taxa annotated at the genus level. Duration of therapy was calculated as the number of days from AT initiation to sample collection. Bar color indicates the differential abundance in each group: dark pink bars represent genera with higher abundance in the sepsis group, light pink bars represent genera with lower abundance in the sepsis group, dark green bars represent genera with higher abundance in the non-sepsis group, and light green bars represent genera with lower abundance in the non-sepsis group.


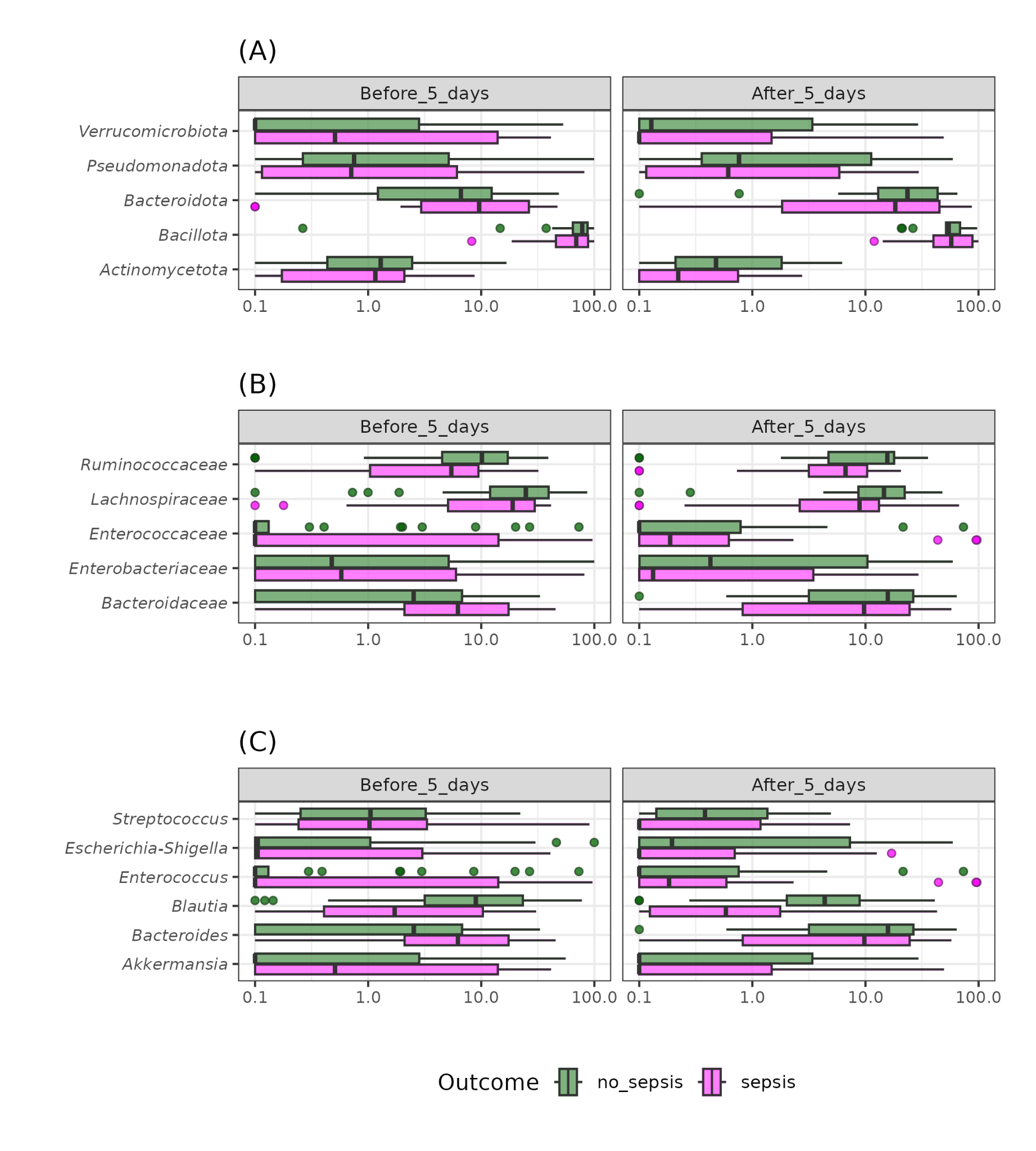


**Supplementary Figure 6. Bar charts showing the top 10 taxa in the gut microbiota between groups over time.** (A) Relative abundance at the phylum level, (B) family level, and (C) genus level in early antibiotic therapy (Before_5_days) and prolonged antibiotic therapy (After_5_days). The sepsis group is represented in purple, and the non-sepsis group in green.

Table S1: Comorbidities among sepsis patients throughout their lifetime and during sepsis episodes.

| SampleID | sepsis_site | Disfunction_type | Infection_site | Comorbidities |
| --- | --- | --- | --- | --- |
| R006A | indefinite | hemodynamic/renal | urinary tract | HTN, DM, AKI |
| R008A | indefinite | hemodynamic/renal | urinary tract/ bacteremia | HFrEF, HTN, T2DM, CVA |
| R009A | indefinite | multiorgan (renal/hemodynamic/SNC) | urinary tract | HTN, T2DM, ACV, HCV, Epilepsy |
| R014A | indefinite | renal/respiratory | urinary tract/ respiratory tract | PD |
| R025A | indefinite | hemodynamic/volemic | indefinite | HTN, T2DM, NHL, Hypothyroidism, CVI, Dementia |
| R026A | indefinite | respiratory | respiratory tract | Psoriatic arthritis, Osteoarthritis, Hypothyroidism, Peptic ulcer, CVA, Chronic hyponatremia |
| R028A | indefinite | volemic/renal | surgical wound/bacteremia | IAM, CKD, Gout |
| R030A | indefinite | hemodynamic/renal | indefinite | HTN, DM, AKI, IHD |
| R033A | urinary | renal | urinary/respiratory tract/bacteremia | CKD, Chagas disease |
| R039A | indefinite | hemodynamic/renal | endocarditis | HTN, Obesity, DRGE |
| R041A | indefinite | hemodynamic | abdominal | HTN, CVA, Thrombolysis, CKD |
| R054A | indefinite | multiorgan (hemodynamic/SNC/respiratory) | respiratory tract | HIV, Aneurysm |
| R056A | pulmonary | multiorgan (renal/hemodynamic/respiratory) | respiratory tract | Malignant neoplasm, Septic arthritis, HTN, DM |
| R057A | indefinite | renal | septic arthritis/urinary tract | HIV, Ankylosing spondylitis, AKI |
| R059A | pulmonary | renal/respiratory | respiratory tract | HTN, ACV, Graves' disease, Psoriatic arthritis |
| R062A | indefinite | volemic | bacteremia/urinary/respiratory tract | HIV, HTN, AKI |
| R068A | indefinite | hemodynamic/renal | indefinite | HTN, TVP/EP, Recurrent erysipelas |
| R071A | indefinite | hemodynamic/respiratory | respiratory tract/bacteremia | HTN, Hypothyroidism, HCV, Prostate adenocarcinoma |
| R072A | skin | multiorgan (renal/hemodynamic/respiratory) | respiratory tract/skin | Cirrhosis, HCV, HCC, Hypertensive gastropathy |
| R074A | indefinite | renal | urinary tract | AKI |
| R075A | indefinite | respitarory | respiratory tract | HIV, HTN, IAM, Asthma |
| R077A | abdominal | multiorgan (hemodynamic/renal/respiratory) | abdominal | HTN, T2DM, CAD, Mitral insufficiency |
| R079A | pulmonary | hemodynamic/volemic | respiratory tract | HFrEF |
| R085A | indefinite | renal | endocarditis | HTN, Erysipela, Dementia |
| R088A | pulmonary | renal/SNC | respiratory tract | Hypothyroidism, DRC, Anemia, Breast neoplasm |
| R092A | pulmonary | renal/SNC/respiratory | respiratory tract | HTN, T2DM, IHD |
| R095A | indefinite | hemodynamic | urinary tract | HTN, RA, Osteoporosis, Fibromyalgia, OA |
| R101A | indefinite | renal/volemic | bacteremia | HTN |
| R102A | indefinite | renal | urinary tract | CKD, COPD |
| R103A | indefinite | multiorgan (hemodynamic/hepatic/renal) | indefinite | HTN, T2DM, CKD, AF, CAD with MI |
| R106A | indefinite | renal | indefinite | Hypothyroidism, T2DM, CKD |
| R058A | indefinite | multiorgan (hemodynamic/respiratory/renal) | indefinite | HTN, DM, Marfan syndrome, Severe aortic insufficiency |
| R081A | indefinite | renal | bacteremia | Mental illness, spondylosis |
| R108A | indefinite | hemodynamic | bacteremia/septic polyarthritis | Polyarthritis, Osteopenia |
| R036A | indefinite | renal | indefinite | HTN, T2DM, COPD, CKD, Asthma |
| R042A | indefinite | hemodynamic/SNC | indefinite | HTN, SAHOS, DRGE |
| R045A | indefinite | renal | bacteremia/urinary tract | MI,COPD,HTN, CHF |
| R070A | indefinite | renal | pyelonephritis | ESRD, T2DM, HTN, CHF, Chronic anemia, Retinopathy, Intercritical tophaceous gout |

HFrEF: Heart Failure with Reduced Ejection Fraction; HTN: Hypertension (also written as High Blood Pressure); T2DM: Type 2 Diabetes Mellitus; CVA: Cerebrovascular Accident; PD: Parkinson’s disease; NHL: Non-Hodgkin Lymphoma; CVI: Chronic Venous Insufficiency; CKD: Chronic Kidney Disease; HIV: Human Immunodeficiency Virus; AKI: Acute Kidney Injury; HCC: Metastatic hepatocellular carcinoma; HCV: Hepatitis C virus; CAD: Coronary artery disease; IHD: Ischemic heart disease; CAD with MI: Coronary Artery Disease with Myocardial Infarction; AF: Atrial Fibrillation; MI: Myocardial infarction; CHF: Congestive heart failure; COPD: Chronic obstructive pulmonary disease; ESRD: End-stage renal disease, TVP/EP: Deep vein tronbosis/ Pulmonary embolism; OA: Osteoarthritis; RA: Rheumatoid arthritis; Obstructive SAHOS: Sleep Apnea-Hypopnea Syndrome.

Table S2: Characteristics of the study population, sample collection timeline, and antimicrobial therapy.

| **Sample**  **ID** | **Age** | **Sex** | **Internation**  **_date** | **Start_**  **ATB** | **Collection**  **_date** | **Collection**  **_time** | **ATB_**  **therapy** | **Stay_**  **days** | **SOFA** | **Outcome** | **Outcome_30_days** | **combi-**  **other** | **BT-comb** |
| --- | --- | --- | --- | --- | --- | --- | --- | --- | --- | --- | --- | --- | --- |
| R001A | 61 | F | 22-8-22 | 22-8-23 | 22-8-25 | 3 | 8 | 10 | 1 | no_sepsis | Alive | Beta_Macro | Beta_Macro |
| R002A | 65 | F | 22-8-24 | 22-8-24 | 22-8-26 | 3 | 7 | 7 | 1 | no_sepsis | Alive | nc | Beta |
| R004A | 94 | F | 22-8-24 | 22-8-28 | 22-8-30 | 3 | 9 | 13 | 1 | no_sepsis | Death | Beta_Amino | Beta_Amino |
| R005A | 64 | F | 22-9-4 | 22-9-4 | 22-9-6 | 3 | 9 | 11 | 0 | no_sepsis | Alive | Beta_Macro | Beta_Macro |
| R006A | 39 | M | 22-9-24 | 22-9-27 | 22-9-30 | 4 | 10 | 77 | 5 | sepsis | Alive | Beta_Glyco | Beta_Glyco |
| R007A | 68 | F | 22-10-1 | 22-10-2 | 22-10-6 | 5 | 9 | 26 | 0 | no_sepsis | Alive | nc | Beta |
| R008A | 70 | M | 22-10-1 | 22-10-3 | 22-10-7 | 5 | 14 | 14 | 5 | sepsis | Alive | nc | Beta |
| R009A | 72 | F | 22-10-5 | 22-10-5 | 22-10-7 | 3 | 9 | 9 | 4 | sepsis | Alive | nc | Beta |
| R011A | 69 | M | 22-10-25 | 22-10-25 | 22-10-28 | 4 | 5 | 7 | 0 | no_sepsis | Alive | Beta_Glyco | Beta_Glyco |
| R012A | 32 | F | 22-10-25 | 22-10-26 | 22-10-28 | 3 | 20 | 21 | 1 | no_sepsis | Alive | Beta_Nitro | Beta_Nitro |
| R013A | 65 | F | 22-10-25 | 22-10-26 | 22-10-30 | 5 | 9 | 12 | 1 | no_sepsis | Alive | nc | Beta |
| R014A | 83 | M | 22-10-26 | 22-10-26 | 22-10-29 | 4 | 9 | 23 | 5 | sepsis | Death | Beta_Amino_  Poli | Beta_Amino_  Poli |
| R015A | 71 | M | 22-11-4 | 22-11-9 | 22-11-11 | 3 | 9 | 9 | 1 | no_sepsis | Alive | Beta_Amino | Beta_Amino |
| R016A | 65 | F | 22-11-2 | 22-11-8 | 22-11-11 | 4 | 5 | 11 | 0 | no_sepsis | Alive | Beta_Macro | Beta_Macro |
| R017A | 61 | F | 22-11-9 | 22-11-9 | 22-11-12 | 4 | 5 | 5 | 0 | no_sepsis | Alive | nc | Beta |
| R018A | 69 | F | 22-11-14 | 22-11-15 | 22-11-18 | 4 | 4 | 5 | 0 | no_sepsis | Alive | nc | Beta |
| R019A | 59 | M | 22-11-16 | 22-11-16 | 22-11-17 | 2 | 3 | 3 | 0 | no_sepsis | Alive | nc | Beta |
| R020A | 41 | M | 22-11-20 | 22-11-20 | 22-11-22 | 3 | 6 | 6 | 0 | no_sepsis | Alive | nc | Beta |
| R021A | 26 | F | 22-11-20 | 22-11-21 | 22-11-23 | 3 | 8 | 13 | 0 | no_sepsis | Alive | Beta_Tetra | Beta_Tetra |
| R022A | 48 | M | 22-11-18 | 22-11-20 | 22-11-26 | 7 | 14 | 18 | 0 | no_sepsis | Alive | Beta_Macro | Beta_Macro |
| R023A | 57 | F | 22-11-23 | 22-11-23 | 22-11-25 | 3 | 4 | 4 | 1 | no_sepsis | Alive | nc | Beta |
| R024A | 52 | M | 22-11-22 | 22-11-22 | 22-11-25 | 4 | 5 | 5 | 1 | no_sepsis | Alive | nc | Beta |
| R025A | 73 | M | 22-11-19 | 22-11-24 | 22-11-26 | 3 | 12 | 23 | 3 | sepsis | Death | Beta_Glyco | Beta_Glyco |
| R026A | 52 | F | 22-11-22 | 22-11-28 | 22-11-30 | 3 | 14 | 21 | 3 | sepsis | Alive | Beta_Amino_  Poli | Beta_Amino_  Poli |
| R027A | 80 | M | 22-11-25 | 22-11-28 | 22-11-30 | 3 | 12 | 18 | 1 | no_sepsis | Alive | Beta_Macro | Beta_Macro |
| R028A | 75 | M | 22-12-11 | 22-12-11 | 22-12-15 | 5 | 17 | 17 | 2 | sepsis | Alive | Beta_Glyco | Beta_Glyco |
| R030A | 47 | F | 22-12-12 | 22-12-12 | 22-12-20 | 9 | 16 | 16 | 4 | sepsis | Alive | Beta_Glyco | Beta_Glyco |
| R031A | 75 | M | 22-12-16 | 22-12-19 | 22-12-23 | 5 | 11 | 14 | 1 | no_sepsis | Alive | nc | Beta |
| R032A | 77 | F | 22-12-16 | 22-12-19 | 22-12-23 | 5 | 6 | 9 | 1 | no_sepsis | Alive | nc | Beta |
| R033A | 68 | F | 22-12-30 | 22-12-30 | 23-1-5 | 7 | 7 | 11 | 4 | sepsis | Alive | Beta_Amino_  Oxazo | Beta_Amino_  Oxazo |
| R034A | 42 | M | 23-1-2 | 23-1-4 | 23-1-6 | 3 | 17 | 30 | 0 | no_sepsis | Alive | nc | Beta |
| R035A | 59 | M | 23-1-8 | 23-1-10 | 23-1-13 | 4 | 7 | 9 | 1 | no_sepsis | Alive | nc | Beta |
| R036A | 84 | M | 23-1-18 | 23-1-18 | 23-1-21 | 4 | 7 | 7 | 4 | sepsis | Alive | Beta_Macro | Beta_Macro |
| R037A | 87 | F | 23-1-15 | 23-1-17 | 23-1-21 | 5 | 8 | 10 | 1 | no_sepsis | Alive | Beta_Amino | Beta_Amino |
| R038A | 40 | F | 23-1-20 | 23-1-20 | 23-1-25 | 6 | 6 | 7 | 1 | no_sepsis | Alive | Beta_Glyco | Beta_Glyco |
| R039A | 44 | F | 23-1-21 | 23-1-22 | 23-1-25 | 4 | 30 | 30 | 6 | sepsis | Alive | nc | nc |
| R041A | 66 | M | 23-2-1 | 23-2-1 | 23-2-8 | 8 | 30 | 30 | 9 | sepsis | Alive | Beta_Macro_  Amino | Beta_Macro_  Amino |
| R042A | 44 | M | 23-2-26 | 23-2-26 | 23-3-6 | 9 | 16 | 31 | 2 | sepsis | Alive | nc | Beta |
| R043A | 75 | F | 23-3-2 | 23-3-2 | 23-3-7 | 6 | 18 | 19 | 1 | no_sepsis | Death | Beta_Glyco | Beta_Glyco |
| R045A | 61 | M | 23-3-2 | 23-3-2 | 23-3-8 | 7 | 10 | 10 | 4 | sepsis | Alive | nc | Beta |
| R046A | 53 | F | 23-3-6 | 23-3-6 | 23-3-8 | 3 | 6 | 6 | 1 | no_sepsis | Alive | nc | Beta |
| R047A | 64 | F | 23-3-26 | 23-3-29 | 23-4-2 | 5 | 8 | 11 | 0 | no_sepsis | Alive | nc | Beta |
| R048A | 87 | M | 23-3-27 | 23-3-27 | 23-4-5 | 10 | 14 | 21 | 0 | no_sepsis | Alive | Beta_Macro | Beta_Macro |
| R049A | 84 | F | 23-4-2 | 23-4-2 | 23-4-5 | 4 | 10 | 10 | 0 | no_sepsis | Alive | nc | Beta |
| R050A | 62 | F | 23-4-4 | 23-4-4 | 23-4-11 | 8 | 14 | 24 | 1 | no_sepsis | Alive | nc | Beta |
| R051A | 63 | M | 23-4-3 | 23-4-3 | 23-4-8 | 6 | 18 | 21 | 1 | no_sepsis | Alive | Beta_Amino | Beta_Amino |
| R052A | 65 | M | 23-4-2 | 23-4-2 | 23-4-8 | 7 | 11 | 30 | 1 | no_sepsis | Alive | nc | Beta |
| R053A | 65 | F | 23-4-18 | 23-4-18 | 23-4-20 | 3 | 13 | 30 | 1 | no_sepsis | Alive | Beta_Glyco | Beta_Glyco |
| R054A | 69 | M | 23-4-2 | 23-4-4 | 23-4-9 | 6 | 14 | 27 | 2 | sepsis | Death | nc | Beta |
| R056A | 22 | F | 23-4-18 | 23-4-18 | 23-4-22 | 5 | 8 | 8 | 5 | sepsis | Alive | Beta_Macro_  Sul | Beta_Macro_  Sul |
| R057A | 84 | M | 23-4-23 | 23-4-23 | 23-4-28 | 6 | 30 | 30 | 2 | sepsis | Alive | Beta_Oxazo | Beta_Oxazo |
| R058A | 54 | M | 23-4-18 | 23-4-23 | 23-4-28 | 6 | 14 | 29 | 6 | sepsis | Alive | nc | Beta |
| R059A | 43 | F | 23-4-23 | 23-4-23 | 23-4-30 | 8 | 20 | 22 | 8 | sepsis | Death | Beta_Macro_  Sul | Beta_Macro_  Sul |
| R060A | 80 | F | 23-4-26 | 23-4-26 | 23-4-30 | 5 | 8 | 9 | 0 | no_sepsis | Alive | Beta_Macro | Beta_Macro |
| R062A | 63 | F | 23-4-28 | 23-4-29 | 23-5-4 | 6 | 30 | 30 | 7 | sepsis | Alive | nc | Beta |
| R064A | 25 | M | 23-5-7 | 23-5-7 | 23-5-11 | 5 | 16 | 17 | 0 | no_sepsis | Alive | Beta_Flu_  Macro | Beta_Flu_  Macro |
| R065A | 23 | F | 23-5-8 | 23-5-8 | 23-5-11 | 4 | 4 | 5 | 0 | no_sepsis | Alive | nc | Beta |
| R067A | 77 | M | 23-5-11 | 23-5-16 | 23-5-17 | 2 | 10 | 23 | 0 | no_sepsis | Alive | nc | Beta |
| R068A | 39 | F | 23-5-16 | 23-5-16 | 23-5-19 | 4 | 13 | 30 | 5 | sepsis | Alive | Flu_Glyco | nc |
| R069A | 49 | F | 23-5-14 | 23-5-15 | 23-5-20 | 6 | 10 | 11 | 1 | no_sepsis | Alive | Beta_Nitro | Beta_Nitro |
| R070A | 87 | M | 23-5-15 | 23-5-15 | 23-5-20 | 6 | 9 | 9 | 8 | sepsis | Alive | Beta_Glyco | Beta_Glyco |
| R071A | 71 | M | 23-5-22 | 23-5-22 | 23-5-25 | 4 | 8 | 10 | 4 | sepsis | Alive | Beta_Macro_  Glyco | Beta_Macro_  Glyco |
| R072A | 63 | M | 23-5-21 | 23-5-22 | 23-5-25 | 4 | 8 | 13 | 4 | sepsis | Alive | Beta_Flu_Tetra_Oxazo | Beta_Flu_Tetra_Oxazo |
| R074A | 74 | M | 23-5-26 | 23-5-29 | 23-6-2 | 5 | 15 | 18 | 4 | sepsis | Alive | nc | Beta |
| R075A | 61 | M | 23-5-29 | 23-5-31 | 23-6-4 | 5 | 10 | 10 | 3 | sepsis | Alive | Beta_Macro | Beta_Macro |
| R077A | 63 | F | 23-6-3 | 23-6-4 | 23-6-6 | 3 | 21 | 23 | 8 | sepsis | Alive | Beta_Tetra_  Nitro | Beta_Tetra_  Nitro |
| R078A | 60 | M | 23-5-28 | 23-5-31 | 23-6-7 | 8 | 30 | 30 | 0 | no_sepsis | Alive | Beta_Flu_Nitro | Beta_Flu_Nitro |
| R079A | 65 | M | 23-6-4 | 23-6-4 | 23-6-9 | 6 | 8 | 9 | 5 | sepsis | Alive | nc | Beta |
| R080A | 32 | M | 23-6-3 | 23-6-5 | 23-6-9 | 5 | 11 | 11 | 1 | no_sepsis | Alive | Beta_Macro | Beta_Macro |
| R081A | 70 | F | 23-6-10 | 23-6-11 | 23-6-14 | 4 | 19 | 20 | 3 | sepsis | Death | Beta_Glyco | Beta_Glyco |
| R083A | 81 | F | 23-6-18 | 23-6-18 | 23-6-22 | 5 | 12 | 20 | 1 | no_sepsis | Alive | Beta_Macro | Beta_Macro |
| R084A | 38 | M | 23-6-20 | 23-6-20 | 23-6-24 | 5 | 7 | 7 | 0 | no_sepsis | Alive | nc | Beta |
| R085A | 61 | M | 23-6-25 | 23-6-27 | 23-6-29 | 3 | 24 | 30 | 2 | sepsis | Alive | Beta_Amino_  Macro_Glyco | Beta_Amino_  Macro_Glyco |
| R087A | 50 | M | 23-6-25 | 23-6-26 | 23-7-3 | 8 | 12 | 17 | 1 | no_sepsis | Alive | Beta_Nitro | Beta_Nitro |
| R088A | 92 | M | 23-7-4 | 23-7-4 | 23-7-9 | 6 | 11 | 17 | 2 | sepsis | Alive | Beta_Flu | Beta_Flu |
| R089A | 79 | F | 23-7-19 | 23-7-19 | 23-7-24 | 6 | 11 | 16 | 1 | no_sepsis | Alive | Beta_Macro | Beta_Macro |
| R090A | 61 | F | 23-7-17 | 23-7-17 | 23-7-24 | 8 | 10 | 10 | 1 | no_sepsis | Alive | Beta_Macro | Beta_Macro |
| R092A | 88 | F | 23-7-16 | 23-7-16 | 23-7-25 | 10 | 12 | 14 | 3 | sepsis | Death | nc | Beta |
| R093A | 68 | M | 23-7-18 | 23-7-18 | 23-7-26 | 9 | 10 | 10 | 1 | no_sepsis | Alive | Beta_Macro | Beta_Macro |
| R094A | 54 | M | 23-7-24 | 23-7-24 | 23-7-27 | 4 | 11 | 14 | 1 | no_sepsis | Alive | Beta_Glyco_  Nitro | Beta_Glyco_  Nitro |
| R095A | 83 | M | 23-7-21 | 23-7-21 | 23-7-28 | 8 | 11 | 12 | 4 | sepsis | Alive | nc | Beta |
| R096A | 71 | F | 23-7-26 | 23-7-26 | 23-7-28 | 3 | 13 | 14 | 1 | no_sepsis | Alive | nc | Beta |
| R101A | 73 | M | 23-7-27 | 23-7-27 | 23-8-3 | 8 | 30 | 30 | 4 | sepsis | Alive | nc | Beta |
| R102A | 72 | M | 23-7-31 | 23-7-31 | 23-8-8 | 9 | 24 | 24 | 4 | sepsis | Alive | Beta_Poli | Beta_Poli |
| R103A | 64 | F | 23-8-6 | 23-8-6 | 23-8-7 | 2 | 30 | 30 | 5 | sepsis | Alive | nc | Beta |
| R106A | 76 | M | 23-7-31 | 23-8-1 | 23-8-10 | 10 | 15 | 15 | 4 | sepsis | Alive | Beta_Glyco | Beta_Glyco |
| R108A | 66 | F | 23-8-12 | 23-8-13 | 23-8-18 | 6 | 30 | 30 | 2 | sepsis | Alive | Beta_Glyco_Rif | Beta_Glyco_Rif |
| R109A | 81 | F | 23-8-17 | 23-8-18 | 23-8-27 | 10 | 13 | 30 | 0 | no_sepsis | Death | nc | Beta |

Table S1: ATB: antimicrobial, ATB_therapy: total time of antimicrobial therapy; SOFA: Sequential Organ Failure Assessment; comb_other: combination of antimicrobial classes; BT-comb: beta-lactam combination with any class of antimicrobial; nc: no combination; Beta: Beta-lactams; Glyco: Glycopeptides; Rif: Rifampicin; Poli: Polymyxins; Macro: Macrolides; Nitro: Nitroimidazoles; Flu: Fluoroquinolones; Amino: Aminoglycosides; Tetra: Tetracyclines; Oxazo: Oxazolidinones.
